# Are static posturography-assisted biofeedback exercises effective in Parkinson's disease?

**DOI:** 10.1055/s-0042-1755325

**Published:** 2022-11-09

**Authors:** Elif Yakşi, Mustafa Fatih Yaşar, Canan Akünal Türel, Muhammed Balcı

**Affiliations:** 1 Abant Izzet Baysal University, Medical Faculty, Department of Physical Medicina and Rehabilitation, Bolu, Turkey.; 2 Abant Izzet Baysal University, Medical Faculty, Department of Neurology, Bolu, Turkey.

**Keywords:** Biofeedback, Psychology, Exercise, Parkinson Disease, Postural Balance, Biorretroalimentação Psicológica, Exercícios, Doença de Parkinson, Equilíbrio Postural

## Abstract

**Background** Parkinson disease (PD) is a progressive condition that causes disorders in movement and balance.

**Objective** To evaluate the effectiveness of static posturography-assisted biofeedback exercises in PD-related balance disorder.

**Methods** We screened 83 patients, 48 of whom were enrolled, and 41 completed the study. The sample was randomized into two groups, one submitted to static posturography-assisted biofeedback exercises and the other, to a conventional exercise program. The patients in the biofeedback group ( *n* = 20) performed biofeedback exercises in addition to conventional balance exercises. Those in the conventional exercise group ( *n* = 21) performed classic balance exercises. Both groups were treated for 20 minutes per session 3 times a week for 6 weeks. The patients were evaluated using the Hoehn and Yahr Scale, the Movement Disorder Society–Unified Parkinson's Disease Rating Scale (MDS-UPDRS), the Berg Balance Scale (BBS), the Tinetti Gait and Balance Assessment (TGBA), the Timed Up and Go Test (TUG), the Tandem Stance Test (TST), a Turkish version of the Stanford Health Assessment Questionnaire (HAQ), and the Beck Depression Inventory (BDI) before and at the end of the treatment.

**Results** No statistically significant differences were observed between the two groups in terms of the MDS-UPDRS, BBS, TGBA, TST, TUG, HAQ, or BDI measurements before and after the treatment ( *p* > 0.05).

**Conclusions** Improved balance parameters were observed following balance training in the patients with PD, although static posturography-assisted biofeedback exercises appeared to provide no additional benefit. However, larger, randomized controlled trials are needed to investigate their effectiveness.

## Resumo

**Antecedentes** A doença de Parkinson (DP) é uma doença degenerativa que causa alterações no movimento e no equilíbrio.

**Objetivo** Avaliar a eficácia dos exercícios com biorretroalimentação assistidos por posturografia estática na alterações do equilíbrio derivadas da DP.

**Métodos** Selecionamos 83 pacientes, 48 dos quais foram incluídos, e 41 completaram o estudo. A amostra foi randomizada e dividida em dois grupos, um submetido a exercícios com biorretroalimentação assistidos por posturografia estática, e outro submetido a um programa de exercícios convencional. Os pacientes do grupo de biorretroalimentação ( *n* = 20) fizeram exercícios com biorretroalimentação e exercícios convencionais de equilíbrio. E o grupo dos exercícios convencionais ( *n* = 21), fez exercícios clássicos de equilíbrio. Ambos os grupos receberam tratamento durante 20 minutos por sessão, 3 vezes por semana, por 6 semanas. Os pacientes foram avaliados antes e depois do tratamento pela Escala de Hoehn e Yahr, Escala Unificada de Avaliação da Doença de Parkinson (EUADP) da Movement Disorder Society (MDS), a Escala de Equilíbrio Berg (EEB), Avaliação de Equilíbrio e Marcha Tinetti (AEMT), o Teste Timed Up and Go (TUG), o Teste de Apoio Tandem (TAT), a versão em turco do Questionário de Avaliação de Saúde (QAS) de Stanford, e o Inventário de Depressão de Beck (IDB).

**Resultados** Não se observaram diferenças estatisticamente significativas entre os dois grupos quanto às medições do EUADP, EEB, AEMT, TAT, TUG, QAS ou IDB realizadas antes e depois do tratamento ( *p* > 0.05).

**Conclusões** Verificou-se uma melhoria dos parâmetros de equilíbrio após os exercícios de equilíbrio nos pacientes com DP, apesar de não ter sido detectado sem nenhum benefício adicional aparente dos exercícios com retroalimentação assistidos por posturografia estática. Contudo, é necessário efetuar ensaios maiores, randomizados e controlados para estudar a sua eficácia.

## INTRODUCTION

Parkinson disease (PD) is a chronic, progressive, degenerative movement disorder, whose incidence increases with age, and characterized by motor and non-motor findings. 1 Cardinal motor findings such as bradykinesia, rigidity, rest tremor, postural instability, and gait dysfunction, and secondary motor symptoms such as bradymimia, dysarthria, and associated movements in the arms may be observed throughout the course of the disease. 2 3 The non-motor findings, including cognitive disorders, dementia, sleep disorders, autonomic symptoms, gastrointestinal dysfunction, and sensory disorders significantly restrict freedom due to their impact on quality of life in PD. 4

Progressive loss of dopaminergic neurons occurs in the basal ganglia, together with a decrease in the speed and angle of movement. Loss of postural reflexes causes balance disturbances such as impaired postural control, rigidity in the extremities, and akinesia. 5 6 Falls, decreased mobility, disability and impairments in quality of life occur as a result of balance disorder. Higher rates of falls or fall-related fractures have been reported in PD elderly patients compared with the non-PD elderly population. 6 Although dopaminergic therapies are the gold standard in the treatment of disorders such as bradykinesia, rigidity, and tremor in PD, sufficient responses are not achieved in the treatment of balance disorder. Rehabilitative measures for balance disorders are therefore gaining importance. 7 There are several exercise applications that provide strategies to improve balance in PD. Strengthening exercises combined with conventional balance exercises, treadmill walking, tai chi, and biofeedback have been shown to improve gait and balance control in PD. 8 Biofeedback is a therapeutic system generally equipped with electronic devices that makes it possible to control or alter the individual's body functions and physiological activities by providing simultaneous biological data. 9 Various publications 10 11 have shown that biofeedback therapy in elderly individuals and in healthy individuals with postural disorder has positive effects on balance by enhancing postural stability and reducing body tremor. However, there has been insufficient research into the effects of biofeedback therapies on balance and the risk of falling in PD.

The basic aim of the present study was to investigate the effects of static posturography-assisted visual and auditory biofeedback therapy on balance and the risk of falls in PD. In addition, the study intended to determine the effects of balance training on daily living activities, perceived safety level, and emotional state.

## METHODS

The present is a prospective, randomized, controlled study. Ethical approval was granted by the Clinical Research Ethics Committee of the Faculty of Medicine of Abant Izzet Baysal University, Turkey (no.: 2018/248). The participants were informed in writing and verbally about the aim of the study, its duration, the therapeutic methods to be employed, and potential side-effects and problems by means of a “Volunteer Information Form” previously developed in the light of the study protocol. Written consent was obtained from all patients agreeing to take part by signing the form.

### Patients

We invited 83 participants (40 women and 43 men) aged between 60 and 80 years who presented to the Physical Medicine and Rehabilitation Clinic of the Abant Izzet Baysal University and were diagnosed with PD between January and June 2019. The inclusion and exclusion criteria are shown in Table 1 .

**Table 1:** Inclusion and exclusion criteria

**Inclusion criteria**
Diagnosis of Parkinson disease,
Age: 60–80 years
Hoehn and Yahr stages 2–3
**Exclusion criteria**
Presence of systemic or neurological diseases capable of causing balance disorder
Postural hypotension
Presence of cardiovascular or musculoskeletal system diseases capable of affecting locomotion
Presence of advanced dementia or mental disability (assessed by the Mini Mental State Examination; cut-off: 23)
Patients who had undergone a rehabilitation program up to 6 months before the study

The power analysis of the study was estimated using the G*Power software package (Franz Faul, Christian-Albrechts University of Kiel, Germany), version 3.1.9.4. We found that to achieve α < 0.05, β = 95%, and 0.75 of effect size according to the TUG scores, the minimum sample size required would be of 20 to identify a statistically significant difference between the 2 groups in terms of repeated measurements according to the study by Maciaszek (2018). 12

The demographic data and clinical characteristics of the participants, such as age, gender, and body mass index (BMI) were recorded. Detailed history was taken, clinical examinations were performed, and existing laboratory and radiological tests were evaluated. The tests and follow-up assessments were performed on average, 2 hours after the patients had taken their morning dose. The patients were instructed to take their Parkinson medication regularly during the study period. All patients were using levodopa-carbidopa, levodopa-carbidopa-entacapone, or a combination of both. All patients were also using 3 mg to 4.5 mg a day of pramipexole hydrochloride as a dopamine receptor agonist, and 1 mg a day of rasagiline mesylate. Amantadine sulfate was used for patients with dyskinesia at a dose of 100 mg to 200 mg a day.

**Figure. 1:**
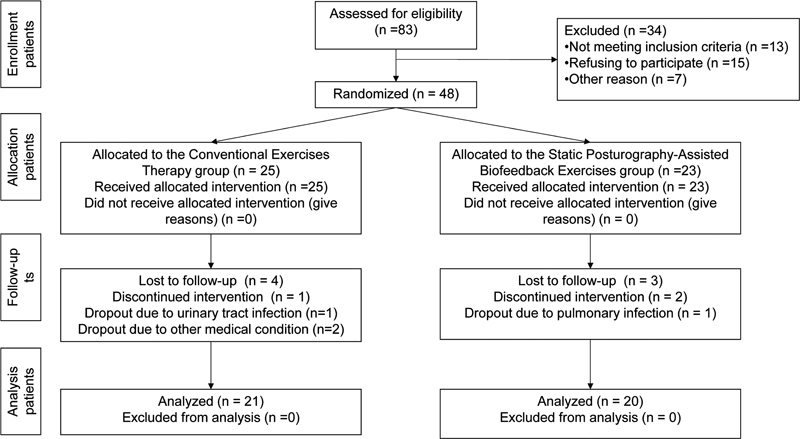
Consolidated Standards of Reporting Trials (CONSORT) flow diagram of the process of inclusion of patients.

The study flow chart is shown in Figure. 1 .

### Evaluation methods

#### *The Hoehn and Yahr Scale*

Prior to treatment, all patients in the present study were classified based on the Hoehn and Yahr Scale, which is widely used to classify PD into five stages. It enables an objective evaluation of the progression of the disease, from stage 0 (no disease symptoms) to stage 5 (the patient is confined to a wheelchair or bed). 13

### The Movement Disorder Society–Unified Parkinson's Disease Rating Scale (MDS-UPDRS)

This is an extensive scale employed for the clinical evaluation of the severity of PD. It consists of four sections including non-motor findings, motor problems, motor findings, and treatment complications. 14 Thirteen motor findings (0 – no finding, or normal, 4- severe finding) in the second section of the scale and comprising the motor problems part were evaluated before and after treatment in this study. Six of the motor examinations (gait, rigidity, arising from a chair, global spontaneity of movement, postural stability, and posture) in the third section of the scale (with scores ranging from 0–normal – to 4–severe) were evaluated before and after treatment.

### Tinetti Balance and Gait Assessment (TGBA)

This scale consists of two sections assessing gait and balance. The first nine questions concern balance, and the following seven concern gait. The total possible score is 28 with 12 points in the gait scale and 16 in the balance scale. Low scores are predictive of balance and gait disorders and an associated risk of falls. 15

### Berg Balance Scale (BBS)

This scale consists of 14 sections, and it measures the ability to maintain balance during voluntary movements and postural changes in the trunk and extremities. The score of each section ranges from 0 (unable to complete the task) to 4 (normal performance). The highest possible score is 56, and total scores from 0 to 20 indicate balance disorder, those between 21 and 40 indicate acceptable balance, and scores ranging from 41 to 56 indicate good balance. 16 The BBS evaluation was performed before and after the treatment.

### Timed Up and Go Test (TUG)

The TUG evaluation was performed before and after the treatment. In this test, the subject is asked to stand up from a chair, walk 3 m, turn around, walk back to the chair, and sit back down, and the time taken to perform this task is recorded in seconds. 17

### Tandem Stance Test (TST)

Tandem stance is a clinical measurement used to assess postural stability and standing balance in a heel-to-toe position on a narrow base of support with a temporal measurement. The individual's ability to maintain static balance is recorded in seconds. 18 The TST was performed before and after the treatment. The mean of three total measurements taken using a chronometer was adopted for each patient.

### Stanford Health Assessment Questionnaire (HAQ)

This questionnaire consists of eight questions designed to assess the effects of an individuals' health status on daily living. It assesses everyday activities such as dressing, hygiene, eating, and walking, and high scores indicate poor health status. 19 The HAQ was applied to all participants before and after the treatment.

### Beck Depression Inventory (BDI)

The BDI is used to evaluate physical, emotional, and cognitive symptoms observed in cases of depression, such as hopelessness, irritability, guilt, fatigue, and weight loss. The total scores range from 0 (no depression) to 63 (severe depression). 20 The BDI scores were evaluated at the beginning and end of the treatment.

### Treatment method

The participants were divided into two groups – conventional exercise (CE) and biofeedback exercise (BE). The first group was prescribed only conventional balance exercises, while the second group was prescribed conventional balance exercises and static posturography-assisted biofeedback exercises. Randomization was performed using a sequential order list with the Microsoft Excel 2013 (Microsoft Corp., Redmond WA, United States) software random number generator function.

All patients were enrolled in a hospital-based rehabilitation program, and they were advised not to take part in an additional exercise program other than their activities of daily living. The patients were informed about the treatment method and potential loss of balance and falls before being taken for treatment. During the study period, the patients were instructed to take their Parkinson medication regularly. Exercises commenced approximately two hours after the patients had taken their morning medications, when PD symptoms such as rigidity, tremor, and bradykinesia were less severe, when easier, freer and faster movements were possible, and when patients felt a subjective improvement.

### Conventional balance exercises

Both groups performed conventional balance exercises (two-leg stance width from wide to narrow, semi-tandem stance, tandem stance, standing on one leg, tandem walking, turning completely around, heel-to-toe stance, standing with the eyes closed, weight shifting, and multidirectional stepping). An exercise program lasting a minimum of 10 minutes was initiated to improve static stance, together with dynamic weight bearing exercises lasting a minimum of 20 minutes. All patients underwent motor and cognitive multitask training in addition to static and dynamic balance exercises. 21 The sessions lasted between 30 and 45 minutes, depending on the level of tolerance of the patients. Patients with balance disorder first underwent balance training in a seated position before progressing to standing exercises, again depending on their level of tolerance. The conventional balance exercise program contained a total of 18 sessions held 3 times a week for 6 consecutive weeks. These were individually tailored based on the patient's tolerance and current motor and sensorial capacities. Each treatment was followed by a 15-minute final phase involving stretching exercises (lumbar extensors, hip flexors, knee flexors, and ankle dorsiflexors) performed three days per week. The patients were supervised by an experienced physiotherapist to avoid falls.

### Balance training with Tetrax

**Figure. 2:**
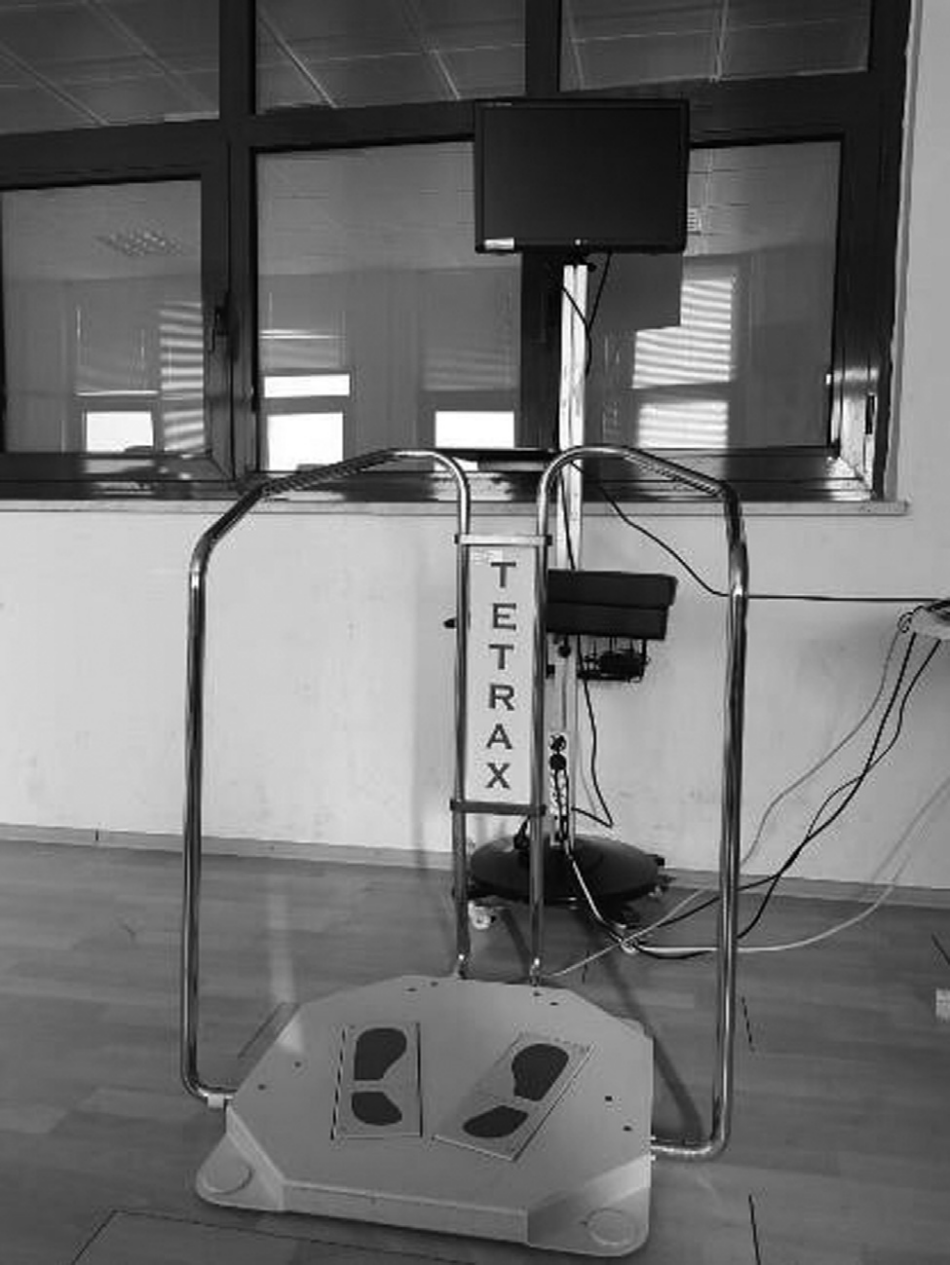
The Tetrax biofeedback system.

The Tetrax (Sunlight Medical Ltd, Tel Aviv, Israel) biofeedback system is a center-of-pressure controlled video game system designed for patients with neurological and orthopedic diseases ( Figure. 2 ). In addition to measuring balance and assessing the risk of falls, the Tetrax device also provides balance training using auditory, visual, and pressure biofeedback. The system contains several games, each designed to focus on a different component of balance. Biofeedback is provided by means of a monitor in front of the patient. Data in this system are obtained using four plates measuring vertical pressure forces from the heel and forefoot. 22 Although sufficient information concerning the clinical use of Tetrax and its feasibility is lacking, it is safer for patients than dynamic balance platforms, which entail a high risks of falls.

In addition to the conventional balance exercises, the BE group performed exercises on the Tetrax, which provided visual and auditory feedback. This balance exercise program involved a total of 18 sessions, each lasting 20 minutes, held 3 times a week for 6 weeks. The level of the therapeutic challenge administered was adjusted based on the size, speed and amount of targets appearing on the screen. In each session, difficulty levels were adjusted on an individual basis depending on the participant's tolerance, current motor and sensorial capacities, abilities, and fatigue levels. 22

**Figure. 3:**
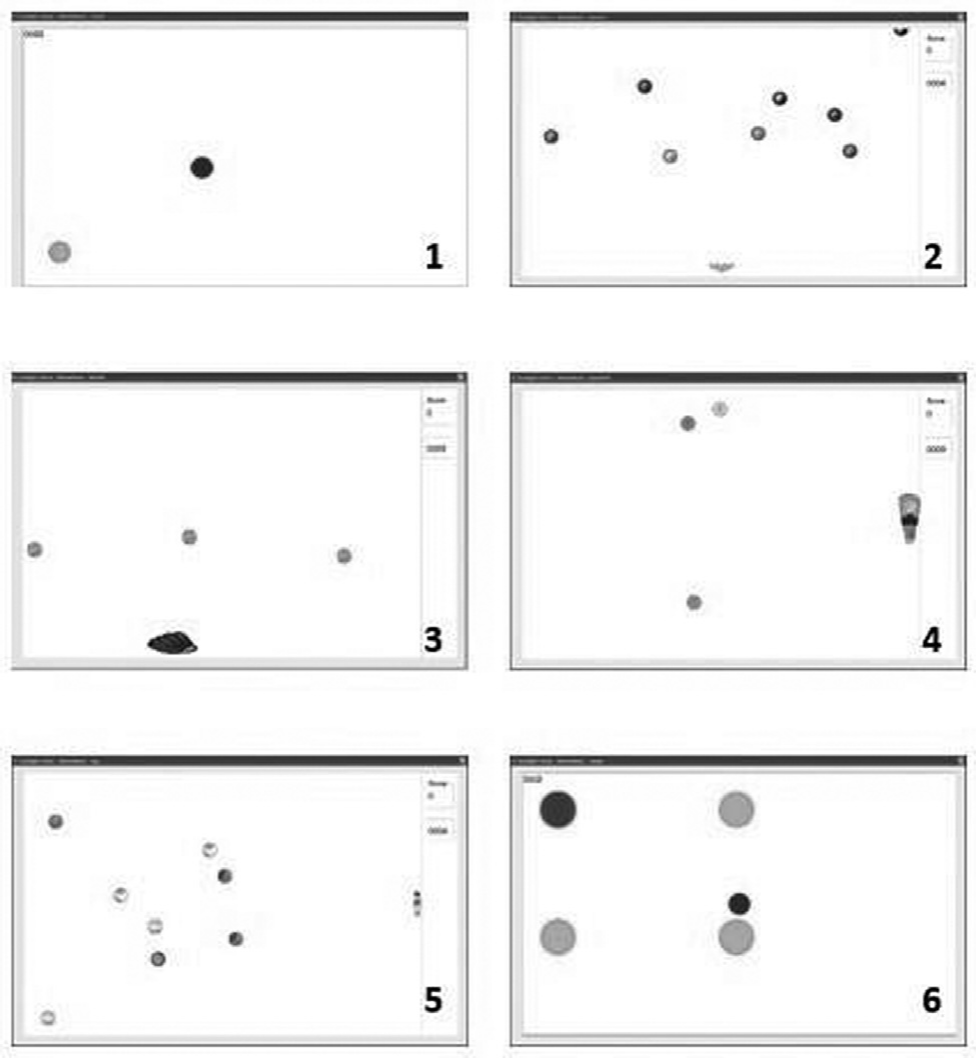
The Tetrax system exercises used in the study. ( **1** ) Catch: the patients try to catch the ball by changing the pressure on their feet while standing on the platform. ( **2** ) Gotcha: the patients try to move the bowling pins by moving their feet left and right to avoid the bowling balls. ( **3** ) Skyball: the patients try to move the baseball glove by moving their feet right and left and try to catch the balls. ( **4** ) Speedball: the patients move the basketball hoop by moving their feet back and forth and try to catch the balls. ( **5** ) Tag: the patients try to avoid the soccer balls by moving their feet back and forth. (6) Target: the patients try to catch the target balls by moving their feet in different directions.

The Catch, Gotcha, Skyball, Speedball, Tag and Target games were used in the biofeedback exercises, as shown in Figure. 3 .

### Statistical analysis

The data obtained were analyzed using the Statistical Package for the Social Sciences (IBM SPSS Statistics for Windows, IBM Corp., Armonk, NY, United States) software, version 21.0. The Mean, standard deviation, median, maximum, and minimum values were calculated for all parameters. The Shapiro-Wilk test was first applied to analyze the normality of the distribution between the two groups. Non-parametric data was analyzed with using the Wilcoxon signed-rank test to investigate difference within the groups, and the paired sample *t* -test was used to compare intragroup changes in normally-distributed variables. Two independent *t* -tests were used to compare the means between the groups for the normally-distributed data. The non-parametric Mann-Whitney U test was used to investigate potential differences between the groups regarding non-parametric variables without normal distribution. The results were evaluated using a 95% confidence interval (95%CI), and values of *p* < 0.05 were considered statistically significant. The Cronbach α coefficient was used to assess the internal consistency of the MDS-UPDRS, TGBA, BBS, HAQ and BDI questionnaires.

## RESULTS

The CE group was composed of 25 participants, and the BE group, of 23 subjects. At the end of the treatment, 4 patients from the CE group and 3 from the BE group were excluded due to failure to attend follow-up consultations or to continue with the treatment; therefore, the study was completed with 41 patients. No side-effects from rhe conventional or biofeedback exercises were observed. The demographic data and clinical characteristics of the study sample are summarized in Table 2 .

**Table 2:** Characteristics and demographic data of the sample

		CE ( *n* = 21)	BE ( *n* = 20)	Total ( *n* = 41)	*p*
Age (years)	Mean ± SD	72.1 ± 6.5	66.8 ± 6.7	69.5 ± 7.0	0.66
	Min.–Max.	60.0/80.0	60.0/79.0	60.0/80.0	
Gender (%)	Male	14 (67%)	8 (40%)	22 (54%)	0.40
	Female	7 (33%)	12 (60%)	19 (46%)	
Body Mass Index (Kg/m ^2^ )	Mean ± SD	28.3 ± 4.2	29.1 ± 4.4	28.8 ± 4.5	0.26
	Min.–Max.	23.3–35.9	24.6–36.1	23.3–36.1	
Hoehn and Yahr stage	Median	2	2	2	0.29
	Min.–Max.	2–3	2–3	2–3	

Abbreviations: BE, biofeedback exercise group; CE, conventional exercise group; Max., maximum; Min., minimum; SD, standard deviation.

Note: α: 0.05.

The values of the Cronbach α coefficient values for the MDS-UPDRS (0,952), TGBA (0,964), BBS (0,940), HAQ (0,962), and BDI (0,968) indicated that these instruments were highly reliable.

No statistically significant difference was observed at initial analysis of patients' clinical characteristics and demographic data ( *p* > 0.05).

The scores on parts II and III of the MDS-UPDRS before and after the treatment are shown in Table 3 , and those of the BBS, TGBA, TST, TUG, HAQ, and BDI are shown in Table 4 .

**Table 3:** A comparison of pre- and posttreatment values within and between the study groups

		CE ( *n* = 21)	BE ( *n* = 20)	*p*
MDS-UPDRS (Part II)*				
**Total score**	Pretreatment	22.1 ± 7.9 (8–37)	22.0 ± 8.5 (9–36)	0.147 ^c^
	Posttreatment	18.6 ± 7.9 (4–34)	17.3 ± 6.6 (6–30)	
	*p*	< 0.001 ^a^	< 0.001 ^a^	
MDS-UPDRS (Part III)**				
**Gait**	Pretreatment	2 (1–3)	1 (1–3)	0.528 ^d^
	Posttreatment	1 (0–3)	1 (0–3)	
	*p*	0.001 ^b^	0.033 ^b^	
**Rigidity**	Pretreatment	1(1–3)	1 (1–2)	0.659 ^d^
	Posttreatment	1 (1–2)	1 (0–2)	
	*p*	0.157 ^b^	0.083 ^b^	
**Arising from chair**	Pretreatment	2 (0–3)	1.5 (1–3)	0.773 ^d^
	Posttreatment	1 (0–3)	1 (0–3)	
	*p*	0.001 ^b^	0.001 ^b^	
**Global spontaneity of** **movement**	Pretreatment	1 (1–2)	1 (1–3)	0.883 ^d^
	Posttreatment	1 (1–2)	1 (0–3)	
	*p*	0.317 ^b^	0.083 ^b^	
**Postural stability**	Pretreatment	1 (1–2)	2 (1–3)	0.560 ^d^
	Posttreatment	1 (0–2)	1 (0–3)	
	*p*	0.002 ^b^	0.002 ^b^	
**Posture**	Pretreatment	1 (1–2)	1 (0–3)	0.415 ^d^
	Posttreatment	1 (0–2)	1 (0–3)	
	*p*	0.157 ^b^	0.083 ^b^	

Abbreviations: BE, biofeedback exercise group; CE, conventional exercise group; MDS-UPDRS, Movement Disorder Society–Unified Parkinson's Disease Rating.

Notes: ^a^ Paired sample *t* -test; ^b^ Wilcoxon signed-rank test; ^c^ independent sample *t* -test; ^d^ Mann-Whitney U test; *Mean ± standard deviation (minimum–maximum); **Median (minimum–maximum).

**Table 4:** Comparison of pre- and posttreatment values within and between the study groups

		CE ( *n* = 21)	BE ( *n* = 20)	*p*
BBS**	Pretreatment	44 (9–54)	44 (15–56)	0.401 ^c^
	Posttreatment	47 (10–56)	51 (35–56)	
	*p*	<0.001 ^a^	<0.001 ^a^	
TGBA**	Pretreatment	20 (8–27)	22 (8–27)	0.119 ^c^
	Posttreatment	23 (10–28)	25 (17–28)	
	*p*	<0.001 ^a^	<0.001 ^a^	
TST**	Pretreatment	4 (0–40)	16 (2–40)	0.979 ^c^
	Posttreatment	30 (1–50)	40 (10–47)	
	*p*	0.001 ^a^	0.003 ^a^	
TUG**	Pretreatment	26 (14–130)	22 (12–50)	0.937 ^c^
	Posttreatment	18 (14–80)	19 (10–30)	
	*p*	<0.001 ^a^	<0.001 ^a^	
HAQ*	Pretreatment	28.8 ± 12.4 (8–57)	22.4 ± 12.5 (2–47)	0.434 ^d^
	Posttreatment	18.9 ± 12.4 (0–50)	10.4 ± 7.4 (0–23)	
	*p*	<0.001 ^b^	<0.001 ^b^	
BDI**	Pretreatment	15 (0–32)	13 (0–44)	0.803 ^c^
	Posttreatment	9 (0–20)	7 (0–25)	
	*p*	<0.001 ^a^	<0.001 ^a^	

Abbreviations: BBS, Berg Balance Scale; BDI, Beck Depression Inventory; BE, biofeedback exercise group; CE, conventional exercise group; HAQ, Stanford Health Assessment Questionnaire; TGBA, Tinetti Balance and Gait Assessment; TST, Tandem Stance Test; TUG, Timed Up and Go Test.

Notes: *Mean ± standard deviation (minimum–maximum); **median (minimum–maximum); ^a^ Wilcoxon signed-rank test; ^b^ paired sample *t* -test; ^c^ Mann-Whitney U test; ^d^ independent sample *t* -test.

The intragroup analysis revealed significant decreases in the scores on parts II and III of the (gait, rising from a chair, and postural stability) of the MDS-UPDRS after the treatment in both groups ( *p* < 0.05). However, the intragroup analysis did not reveal significant differences in the posttreatment scores of part III (posture, global spontaneity of movement and rigidity) compared with the pretreatment scores in either group ( *p* > 0.05).

The scores of both study groups on each of the assessment instruments used before and after the treatment were compared, and no statistically significant differences were found ( *p* > 0.05).

## DISCUSSION

The basic hypothesis of the present study was that adding auditory and visual biofeedback methods to classic balance exercises would contribute to improve postural control and balance parameters in PD patients. The results showed a significant improvement in the parameters after the treatment in both groups, but no statistically significant differences were observed between the groups.

Biofeedback therapies have been used for many years in diseases in addition to medical treatment and rehabilitation techniques. They are also an alternative therapeutic option against the side effects of and lack of response to pharmacological therapies. 23 The development of computer-based systems in particular has caused a spread in the use of biofeedback techniques in hospital-based and home-based neurorehabilitation programs. Biofeedback therapies are now frequently employed in neurological diseases, particularly in stroke rehabilitation programs. Walker et al. 24 investigated the effects on balance parameters of visual biofeedback therapy in addition to conventional exercises in acute ischemic stroke patients. The study involved two experimental groups and one control group, and a significant improvement was observed all of them, but no significant differences were observed. 24 The data from the study by Walker et al. 24 are compatible with those of the present study. In one review study, 25 the authors reported that force platform-assisted feedback exercises improved stance symmetry, but provided no significant improvement in balance parameters or functional independence. In contrast to this study, Maciaszek 12 reported more effective improvement in static and dynamic balance parameters in elderly stroke patients undergoing posturographic platform biofeedback compared with a control group submitted to conventional exercises. The duration and severity of stroke, physical, mental, psychological and social variations, and accompanying diseases can alter the response to biofeedback therapy. In contrast to the post, stroke period when the neurological findings of the patients follow a stable course, in PD, the disease is progressive and causes increasing neuronal damage, which may result in different responses to treatment.

Few studies have investigated the effects on balance parameters in PD of posturography-assisted biofeedback exercises. In the study by Balcı et al., 26 four PD patients underwent static posturography-assisted biofeedback exercises for three weeks, and no significant improvement in the balance parameters was observed. 26 In a study by Mirelman et al. 27 seven pD patients were submitted to audio feedback integrated into active exercise programs over a period of six weeks, and the authors observed a significant improvement in the balance parameters. The biofeedback therapies in those studies were integrated into concurrent active exercise applications, while in the present study the biofeedback was applied with minimal body movements being senses on a fixed platform with a pressure sensor and the resulting data appearing on the screen in the form of visual and auditory signals. The fact that static and dynamic balance exercises were combined with biofeedback may have resulted in positive effects on the balance parameters.

Findings concerning the efficacy of biofeedback in PD are inconsistent. Pompeu et al. 28 examined the effectiveness of biofeedback therapies on motor functions in PD. They applied conventional balance exercises and global exercises to one group, and feedback exercises assisted by Wii (Nintendo Co., Ltd., Kyoto, Japan) in addition to global exercises to the other group. A significant improvement in the scores on part II of the MDS-UPDRS was observed in both groups, but no differences between them in terms of effectiveness were found. 28 Yen et al. 29 compared the effectiveness of virtual reality-augmented balance training and conventional balance exercises in PD. They 29 concluded that both groups developed sensory integration for postural control, but that no additional benefit in terms of postural control was achieved on the sensory organization tests. The findings of these studies 28 29 are compatible with those of the present study.

Although no significant effect of the biofeedback exercises on balance parameters was determined in the present study, all patients reported enjoying the training program. The therapists reported finding the static posturography device safer than the dynamic balance devices, and that patient participation was higher in that group. Conventional exercises employed to improve postural stability and in the treatment of balance disorders in PD are both safe and effective. Such exercises can be performed in any rehabilitation center or home-based rehabilitation programs with no additional cost. They are also more practicable than other methods since they require no extra equipment.

The main limitation of the present study is the inadequate sample size due to the low incidence of PD. Another limitation is that the patients were in different clinical stages (stages 2 and 3 on the Hoehn and Yahr scale). Greater neuronal loss in patients in advanced stages may have resulted in lower therapeutic success. In addition, the absence of a group only undergoing static posturography-assisted biofeedback exercises prevented us from examining the effects of this method in isolation. Balance impairment in PD may be derived from the extrapyramidal system, or it may be associated with non-motor findings resulting from the involvement of non-dopaminergic regions. The fact that non-motor symptoms as a potential cause of balance impairment were not investigated in detail is another limitation of the study. In addition, the application of a standard biofeedback program to all participants and the absence of individualized programs may also have adversely impacted the success of the treatment.

In conclusion, although static posturography-assisted biofeedback exercises yielded high patient participation and satisfaction, no additional benefit of these exercises on balance parameters was detected. Subsequent randomized controlled studies that are more extensive are required to investigate the effectiveness of static posturography-assisted biofeedback exercises.
